# The regulation of insulin receptor/insulin-like growth factor 1 receptor ratio, an important factor for breast cancer prognosis, by TRIP-Br1

**DOI:** 10.1186/s13045-022-01303-6

**Published:** 2022-06-16

**Authors:** Thi Ngoc Quynh Nguyen, Samil Jung, Hai Anh Nguyen, BeomSuk Lee, Son Hai Vu, Davaajargal Myagmarjav, Hye Hyeon Eum, Hae-Ock Lee, Taeyeon Jo, Yeongseon Choi, Myeong-Sok Lee

**Affiliations:** 1 Department of Biological Science, Sookmyung Women’s University, Cheongpa-ro 47-gil 100, Yongsan-gu, Seoul, 14310 South Korea; 2 Department of Biomedicine and Health Sciences, Graduate School, The Catholic University of Korea, Seoul, South Korea; 3 Department of Microbiology, College of Medicine, The Catholic University of Korea, Seoul, South Korea

**Keywords:** Breast cancer, IR, IGF1R, TRIP-Br1, NEDD4-1

## Abstract

Much higher risk of cancer has been found in diabetes patients. Insulin receptor (IR) and insulin-like growth factor 1 receptor (IGF1R) have been extensively studied in both breast cancer and diabetes therapies. Interestingly, a recent study proposed that IR/IGF1R ratio is an important factor for breast cancer prognosis. Women with higher IR/IGF1R ratio showed poor breast cancer prognosis as well as hyperinsulinemia. Here, we propose a novel mechanism that oncogenic protein TRIP-Br1 renders breast cancer cells and insulin deficient mice to have higher IR/IGF1R ratio by positively and negatively regulating IR and IGF1R expression at the protein level, respectively. TRIP-Br1 repressed IR degradation by suppressing its ubiquitination. Meanwhile, TRIP-Br1 directly interacts with both IGF1R and NEDD4-1 E3 ubiquitin ligase, in which TRIP-Br1/NEDD4-1 degrades IGF1R via ubiquitin/proteasome system. TRIP-Br1-mediated higher IR/IGF1R ratio enhanced the proliferation and survival of breast cancer cells. In conclusion, current study may provide an important information in the regulatory mechanism of how breast cancer cells have acquired higher IR/IGF1R ratio.

**Supplementary Information:**

The online version contains supplementary material available at 10.1186/s13045-022-01303-6.

## To the editor,

The relationship between breast cancer and diabetes has been extensively studied. Women with diabetes are at a greater risk of developing breast cancer than those without diabetes [1–3]. Interestingly, a recent study suggested that the IR/IGF1R ratio is a key factor in breast cancer prognosis, by evaluating IR/IGF1R ratio in over 500 patients with breast cancer [4]. They showed that breast cancer patients with a higher IR/IGF1R ratio due to elevated IR expression not only have hyperinsulinemia but are also more susceptible to enhance tumorigenesis [4]. It was reported that TRIP-Br1 plays an important role in diabetes [5]. In addition, TRIP-Br1 is one of the most up-regulated genes in both type 1 and type 2 diabetes [6]. Moreover, high levels of TRIP-Br1 were detected in various subtypes of breast cancer [7, 8]. In this study, we explored the regulatory mechanism of TRIP-Br1 in controlling the IR/IGF1R ratio in breast cancer cells.

**Fig. 1:**
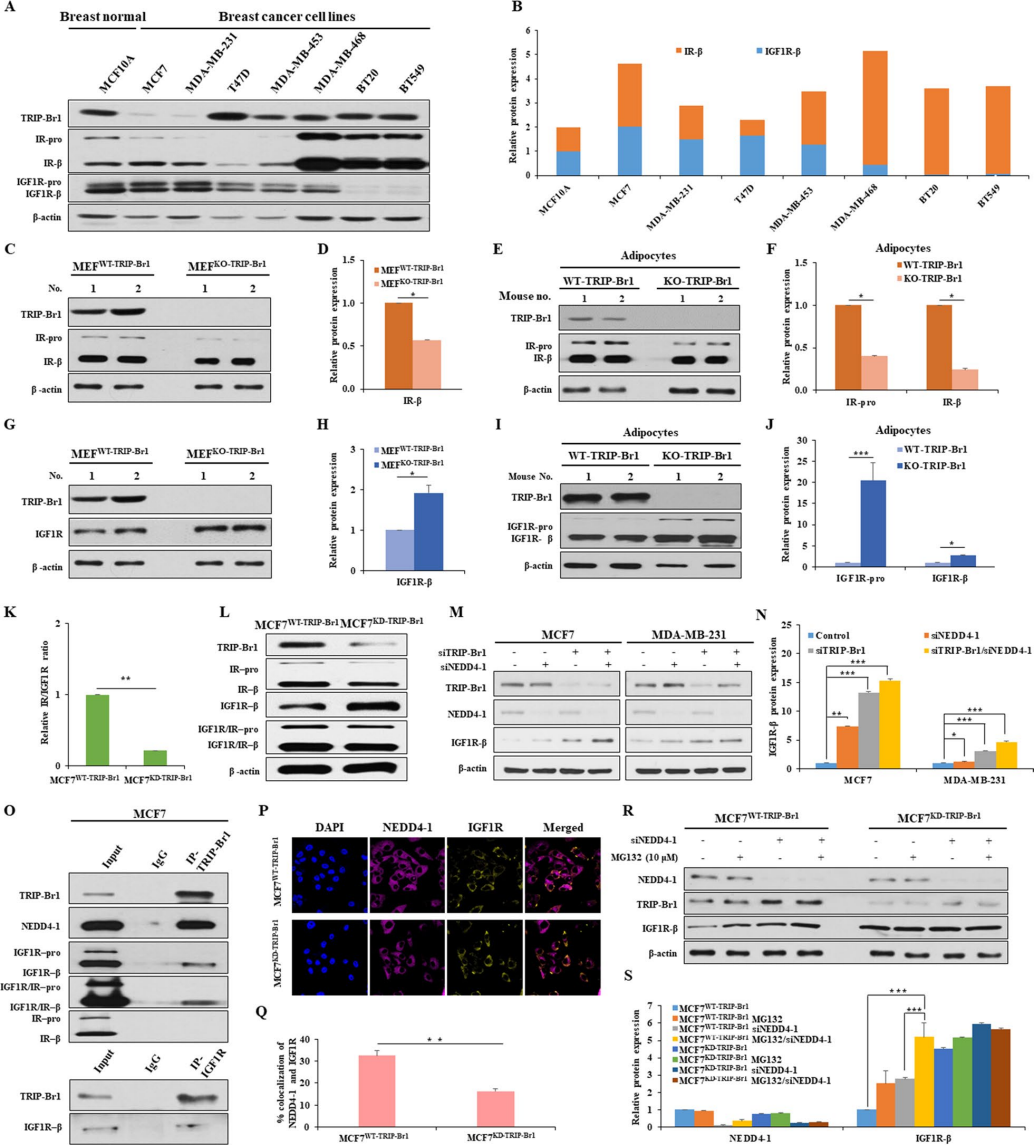
IR/IGF1R ratio is regulated by TRIP-Br1 in breast cancer cells. **A** Expression levels of TRIP-Br1, IGF1R, and IR were checked in breast normal and cancer cell lines by western blotting. β-actin was used as a loading control. **B** IR/IGF1R ratio was quantified using ImageJ. **C**, **D** Endogenous IR expression was assessed in MEF cells isolated from TRIP-Br1 wild-type (MEF^WT-TRIP-Br1^) or knockout mice (MEF^KO-TRIP-Br1^), as mentioned in the Materials and Methods (*n* > 3) (Additional file 3). **E**, **F** The IR protein levels from adipocytes tissue collected from TRIP-Br1 wild-type or knockout mice were evaluated by western blotting (*n* = 3). **G**, **H** The TRIP-Br1 and IGF1R expression levels were measured in MEF^WT-TRIP-Br1^ or MEF^KO-TRIP-Br1^ cells by western blotting. **I**, **J** The protein levels of TRIP-Br1 and IGF1R were checked in adipocytes tissue collected from TRIP-Br1 wild-type or knockout mice (*n* = 3). **K** The relative IR/IGF1R ratio is shown in MCF7 stable cell lines with TRIP-Br1 wild-type (MCF7^WT-TRIP-Br1^) and knock-down (MCF7^KD-TRIP-Br1^) cells. **L** The indicated protein levels were evaluated by western blotting. The expression of IGF1R and IR was co-analyzed using a co-antibody that recognizes both IGF1R and IR. **M**, **N** TRIP-Br1 or NEDD4-1 silencing RNA (siTRIP-Br1 and siNEDD4-1) were transfected into indicated cell lines and IGF1R expression was analyzed by using a western blot analysis (*n* > 3). **O** The interaction between IGF1R and TRIP-Br1 was determined by using co-immunoprecipitation assay. **P**, **Q** The representative images of NEDD4-1 and IGF1R expression were observed using a confocal microscope. The co-localization between NEDD4-1 and IGF1R was measured by counting over 50 cells in ImageJ. Data are presented as the mean ± SD (*n* > 50). **R**, **S** Cells were transfected with siNEDD4-1 in the absence or presence of MG132 (10 μM) for 24 h and subjected to western blotting (*n* = 3). The quantification results are presented as the mean ± SD (*p < 0.05; **p < 0.01; ***p < 0.005)

IR and IGF1R expression levels were normalized in MCF10A to compare the IR/IGF1R ratio in breast cancer cell lines. In particular, cancer cell lines with very high levels of TRIP-Br1 showed a much higher IR/IGF1R ratio than the others (Fig. 1A, B). Our data revealed that MEF^WT-TRIP-Br1^ cells showed a higher IR expression levels, compared to MEF^KO-TRIP-Br1^ cells (Fig. 1C, D) (Additional file 1: Fig. S1A–C). TRIP-Br1 wild-type mice also showed approximately 2–4-fold higher IR expression levels in adipocyte and heart tissue samples compared with TRIP-Br1 knockout mice (Fig. 1E, F) (Additional file 1: Fig. S1D–F). Further study revealed that TRIP-Br1 increased IR protein level by suppressing proteasome-mediated degradation of IR (Additional file 1: S1G, H). Interestingly, IR silencing elevates IGF1R expression, resulting in a lower IR/IGF1R ratio (Additional file 1: Fig. S1I, J). On contrast, TRIP-Br1 negatively affects IGF1R expression, eventually increasing the IR/IGF1R ratio (Fig. 1G, H). While TRIP-Br1 overexpression significantly decreased IGF1R expression, TRIP-Br1 silencing greatly increased IGF1R expression (Additional file 1: Fig. S2A–F). Furthermore, TRIP-Br1 knockout mice showed elevated IGF1R in adipocytes (~ 20-fold at IGF1R-pro and ~ two-fold at IGF1R-β) and the heart (~ two-fold) compared to control mice (Fig. 1I, J) (Additional file 1: Fig. S2G, H). In addition, MCF7^WT-TRIP-Br1^ cells showed the higher IR but lower IGF1R expression at protein level, resulting in a high IR/IGF1R ratio, compared with MCF7^KD-TRIP-Br1^ cells (Fig. 1K, L). However, TRIP-Br1 does not affect IR and IGF1R at the transcriptional level (Additional file 1: Fig. S2I).

Interestingly, the IGF1R expression levels greatly increased when TRIP-Br1 and/or NEDD4-1 E3 ligase were silenced (Fig. 1M, N). Co-immunoprecipitation experiments reveled a direct interaction between TRIP-Br1 and IGF1R, as well as NEDD4-1, while no direct interaction was observed between TRIP-Br1 and IR. This data imply that TRIP-Br1 may serve as an adaptor protein to bring NEDD4-1 close enough to IGF1R (Fig. 1O). Co-immunofluorescence experiments support the notion that TRIP-Br1 facilitates NEDD4-1/IGF1R interaction (Fig. 1P, Q). We also show that TRIP-Br1/NEDD4-1 degraded IGF1R mainly through the proteasome/ubiquitination pathway rather than through a lysosomal pathway (Fig. 1R, S) (Additional file 1: Fig. S3C–H and Additional file 2).

**Fig. 2:**
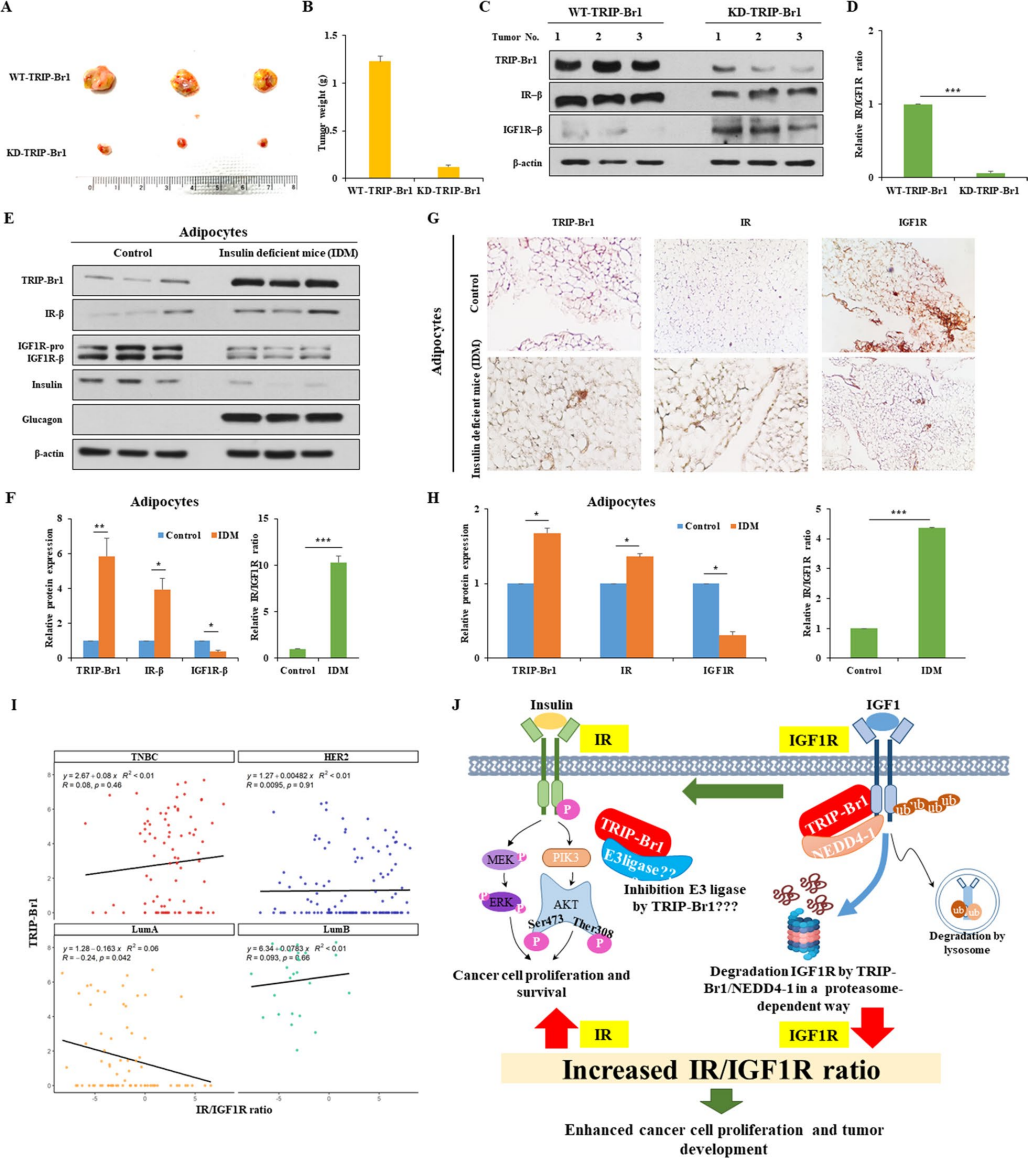
Enhanced tumor formation and growth is associated with a higher IR/IGF1R ratio resulting from TRIP-Br1 expression. **A**, **B** MCF7^WT-TRIP-Br1^ and MCF7^KD-TRIP-Br1^ cells were injected into nude mice. The tumors were collected and photographed. Scale bar, 1 cm. Tumor weight was measured after mice resection (*n* > 6). **C**, **D** Tumor was collected and subjected to western blotting. The relative IR/IGF1R ratio is presented as the mean ± SD (*n* > 6; ***p < 0.005). **E**, **F** Tissue samples from the adipocytes were collected from 5-week-old insulin-producing mice (control) or insulin-deficient mice (IDM). The tissues were used to assess the levels of TRIP-Br1, IR, and IGF1R by western blot analysis, in which insulin and glucagon were used as controls (*n* > 3). The relative IR/IGF1R ratio is shown. The quantification results are presented as the mean ± SD (*p < 0.05; **p < 0.01; ***p < 0.005). **G**, **H** Representative images of IHC analysis show the expression levels of TRIP-Br1, IR, and IGF1R in the adipocytes of control or IDM groups. The expression levels of TRIP-Br1, IR, and IGF1R are presented as the mean ± SD (*n* > 3). The relative IR/IGF1R ratio is shown. (*p < 0.05; ***p < 0.005). **I** The correlation of TRIP-Br1 and IR/IGF1R ratio in four subtypes of breast cancer. Each dot represents a single cell. **J** Summary model shows the regulation of the IR/IGF1R ratio by TRIP-Br1 in breast cancer cells

Animal experiments indicated that TRIP-Br1 enhanced tumor progression, where a high IR/IGF1R ratio was detected (Fig. 2A, B) (Additional file 1: Fig. S4A, B). In agreement with the in vitro observations, an approximately ten-fold higher IR/IGF1R ratio, due to the higher IR but lower IGF1R, was detected in wild-type TRIP-Br1 producing cancer cells grown in nude mice (Fig. 2C, D). Our extended study showed a similar effect of TRIP-Br1 on the IR/IGF1R ratio in insulin-deficient mice mimicking patients with diabetes, implying that TRIP-Br1 may be a potential target for the treatment of both diabetes and breast cancer (Fig. 2E–H) (Additional file 1: Fig. S5A–D).

We further explored the relationship between TRIP-Br1 and the IR/IGF1R ratio by analyzing 317 tumor single cells from 11 breast cancer patients. They were divided into four representative subtypes as shown in GSE75688 datasets (Additional file 1: Table S1) [9]. Triple-negative breast cancer (TNBC) tumor cells showed a positive correlation between the TRIP-Br1 expression and the IR/IGF1R ratio but luminal A (LumA) subtypes cells revealed the opposite results (Fig. 2I) (Additional file 1: Fig. S6A, B). However, bioinformatics analysis (http://timer.cistrome.org/) from the database, with as many as 568 patients, showed that LumA cells show an inverse relationship between TRIP-Br1 and IGF1R expression, similar to our in vitro results (Additional file 1: Fig. S6C). Our bioinformatics analysis revealed that TRIP-Br1 positively correlated with the IR/IGF1R ratio but inversely with survival time in breast cancer patients (*n* = 152). However, no significant relationship was observed in lung (*n* = 396) or liver cancer (*n* = 130) (Additional file 1: Fig. S7). This implies that TRIP-Br1 may be a breast cancer-specific oncogenic adaptor protein.

In conclusion, our findings provide valuable insights on the regulatory mechanisms of the IR/IGF1R ratio. TRIP-Br1-mediated higher IR/IGF1R ratio increased the survival rate of breast cancer cells, resulting in a worse prognosis for breast cancer patients. Therefore, the TRIP-Br1-mediated IR/IGF1R ratio appears to be a predictive factor for the prognosis and progression of cancer. Summary model is shown in Fig. 2J (Additional file 2).

## Supplementary Information

**Additional file 1:** Supplementary Figures.

**Additional file 2:** Supplementary Introduction and Results.

**Additional file 3:** Materials and Methods.

## Data Availability

All the data supporting the findings of this study within the article, and its additional files are available from the corresponding author upon reasonable request.

## Abbreviations

CQ: Chloroquine
EMT: Epithelial-mesenchymal transition
IDM: Insulin deficient mice
IGF1R: Insulin-like growth factor 1 receptor
IR: Insulin receptor
LumA: Luminal A (ER^+^/HER^−^)
MG132: *N*-Benzyloxycarbonyl-l-leucyl-l-leucyl-l-leucinal
NEDD4-1: Neural precursor cell expressed developmentally downregulated protein 4-1
TNBC: Triple-negative breast cancer (ER^−^/HER^−^)
TRIP-Br1: Transcriptional regulator interacting with the PHD-bromodomain 1
