# Supplementary material for: The regulation of insulin receptor/insulin-like growth factor 1 receptor ratio, an important factor for breast cancer prognosis, by TRIP-Br1
Source: J Hematol Oncol. 2022 Jun 16;15:82. doi: 10.1186/s13045-022-01303-6 (PMC9204904; doi:10.1186/s13045-022-01303-6)
Supplement: Supplementary file 1 — Additional file 1: Supplementary Figures. [file 13045_2022_1303_MOESM1_ESM.docx]

**Supplementary Figures**

**Supplementary Figure 1.**

**
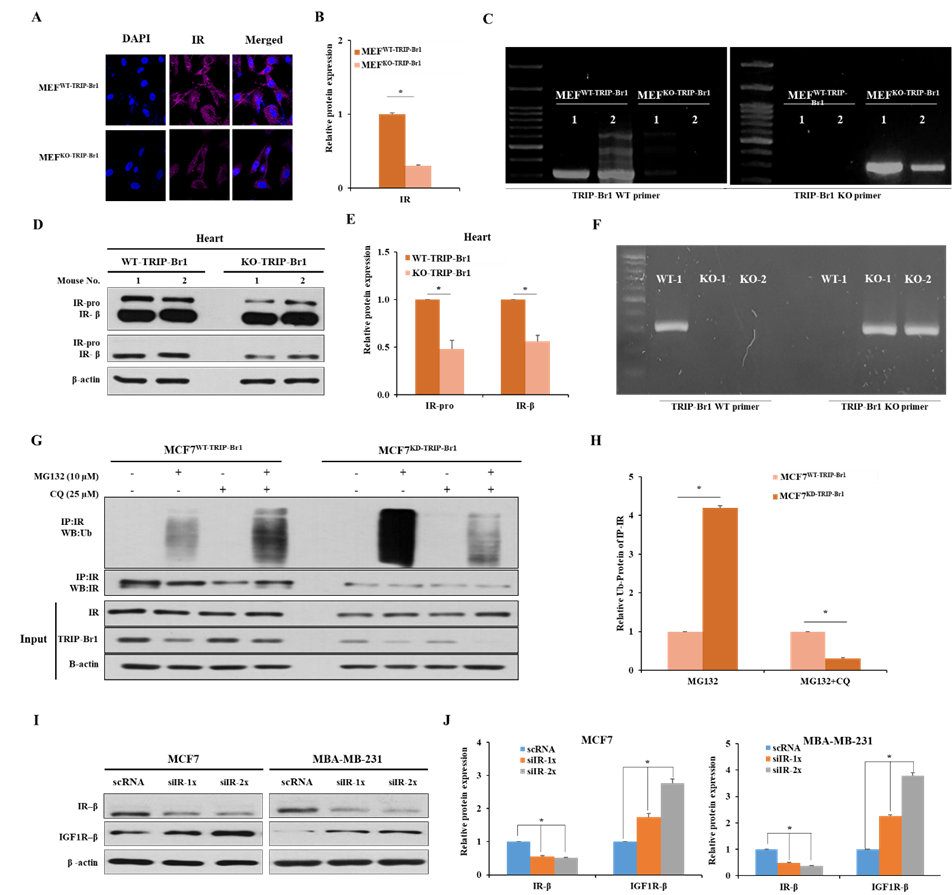
**

**Supplementary Figure 1. Supplementary results for the positive impact of TRIP-Br1 on IR expression. A-B** Expression level of IR in MEF^WT-TRIP-Br1^ or MEF^KO-TRIP-Br1^ cells. Quantification is shown as the mean ± SD based on three independent experiments (n = 3). Asterisk (*) indicates statistically significant difference at p < 0.05. **C** MEF^WT-TRIP-Br1^ and MEF^KO-TRIP-Br1^ were confirmed by PCR [1]. TRIP-Br1 wild-type (WT) forward/reverse primers: 5’-CCATCCCCAGCATCAAATACACCA-3’/5’-CTCCCGCTTGCGCTTCAGACCTT-3’; TRIP-Br1 knockout (KO) forward/reverse primers: 5’-CCATCCCCAGCATCAAATACACCA-3’/5’-CATAGCCTGAAGAACGAGAT-3’.**D-E** The IR protein levels from heart tissue collected from TRIP-Br1 wild-type or knockout mice were evaluated by western blotting (n = 3; *, p < 0.05). β-actin was used as the loading control. **F** TRIP-Br1 wild-type (WT) and knockout (KO) mice were confirmed using the same primers [1]. **G-H** MCF7^WT-TRIP-Br1^ and MCF7^KD-TRIP-Br1^ cells were treated with MG132 (10 µM) and/or CQ (25 µM) for 24 h. Endogenous IR was immunoprecipitated with anti-IR antibody. Ubiquitinated IR was analyzed with anti-Ub by employing western blot. Data are presented as the mean ± SD (n > 3; *, p < 0.05). **I-J** IR was knocked down using IR silencing RNA (siIR). scRNA was used as a control. The indicated protein levels were evaluated by western blot analysis. The results are presented as the mean ± SD (n = 3; *, p < 0.05).

**Supplementary Figure 2.**

**
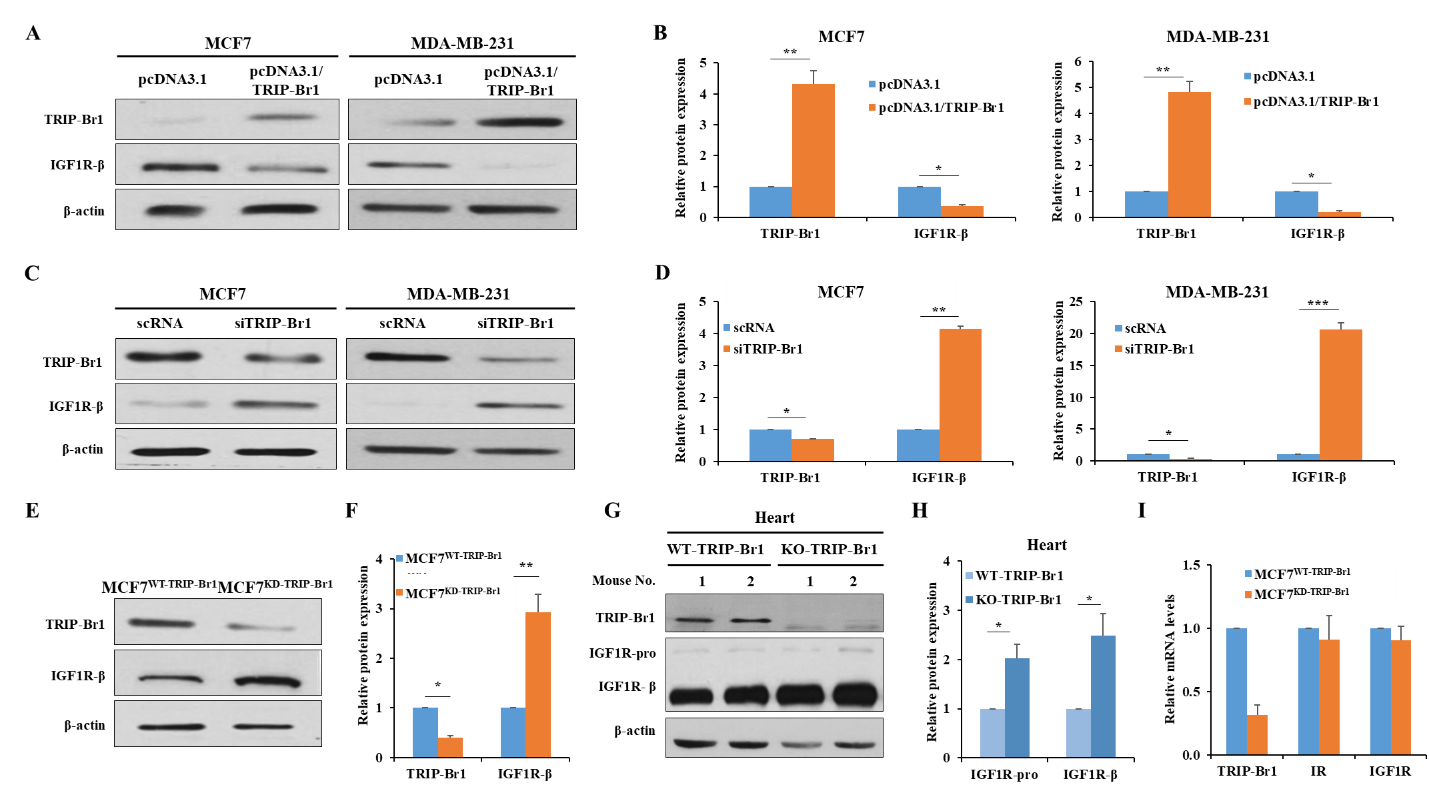
**

**Supplementary Figure 2. Supplementary results for the negative effect of TRIP-Br1 on IGF1R expression. A-B** TRIP-Br1 was overexpressed by transfecting pcDNA3.1/TRIP-Br1 into MCF7 and MDA-MB-231 cells, in which pcDNA3.1 empty was used as a control. Cells were collected and prepared for western blot analysis. The results of western blotting were quantified using ImageJ. Analysis was conducted in triplicate and data are presented as the mean ± SD (n = 3; *, p < 0.05;**, p < 0.01). **C-D** TRIP-Br1 silencing RNA (siTRIP-Br1) was transfected into MCF7 and MDA-MB-231 cells, in which scrambled RNA (scRNA) was used as a non-silencing control. Data are presented as the mean ± SD (n = 3; *, p < 0.05; **, p < 0.01; ***, p < 0.005). **E-F** TRIP-Br1 and IGF1R expression levels in MCF7^WT-TRIP-Br1^ and MCF7^KD-TRIP-Br1^ cells. Data are presented as the mean ± SD (n = 3; *, p < 0.05;**, p < 0.01). **G-H** The protein levels of TRIP-Br1 and IGF1R in heart tissue collected from TRIP-Br1 wild type or knockout mice. The results are presented as the mean ± SD (n = 3; *, p < 0.05). **I** The mRNA expression level of TRIP-Br1, IR and IGF1R in MCF7^WT-TRIP-Br1^ an MCF7^KD-TRIP-Br1^.

**Supplementary Figure 3.**

**
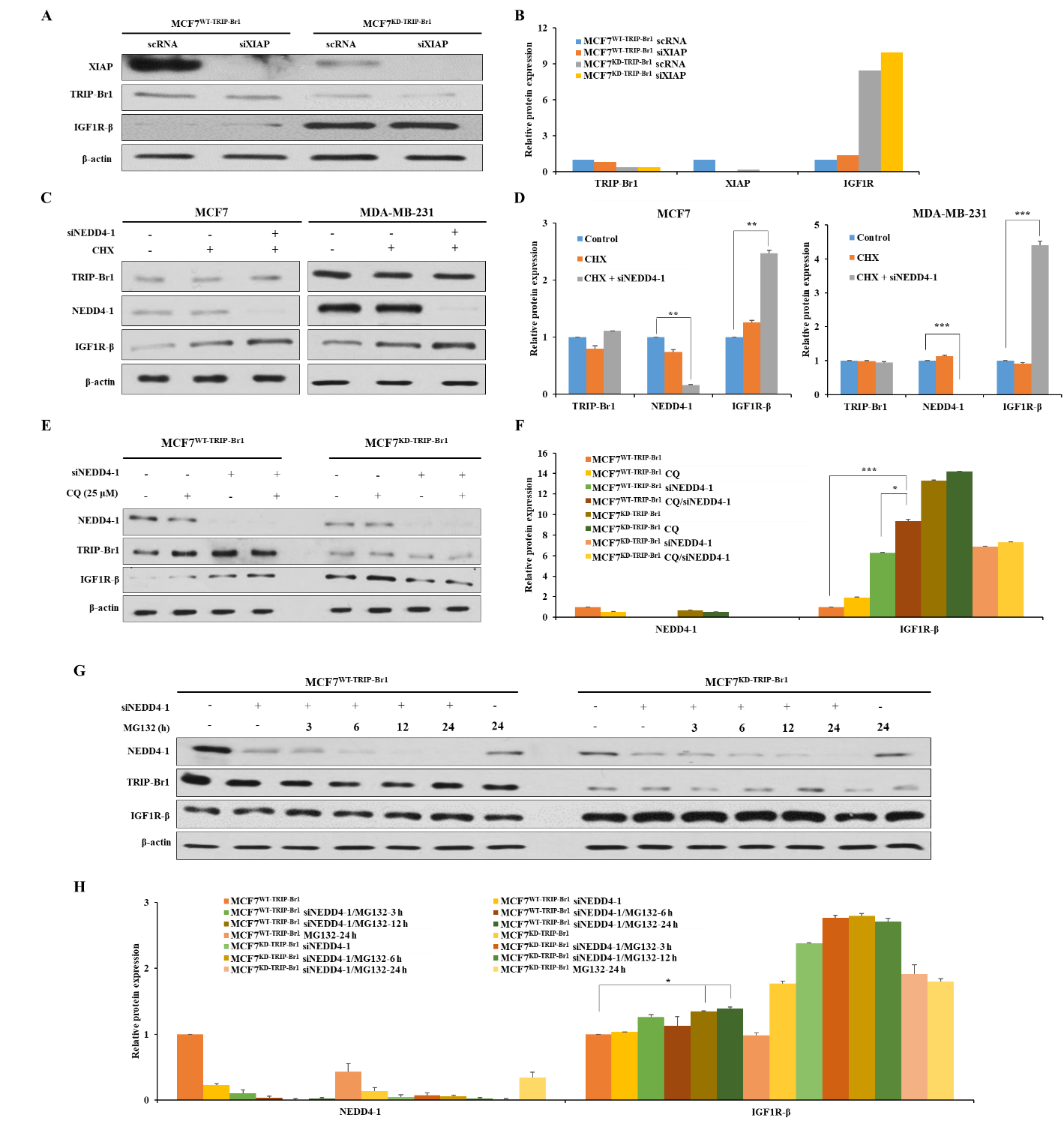
**

**Supplementary Figure 3. Supplementary results for the TRIP-Br1/NEDD4-1 mediated IGF1R degradation. A-B** XIAP silencing RNA (siXIAP) were transfected into MCF7^WT-TRIP-Br1^ and MCF7^KD-TRIP-Br1^ cells and IGF1R expression was analyzed by using a western blot analysis. **C-D** NEDD4-1 was knocked down by siNEDD4-1 in the presence of 200 mM CHX and the indicated proteins were subjected to western blot analysis. Data are presented as the mean ± SD (n > 3; **, p < 0.01; ***, p < 0.005). **E-F** MCF7^WT-TRIP-Br1^ and MCF7^KD-TRIP-Br1^ cells were transfected with siNEDD4-1 with or without CQ (25 µM) for 24 h. Total cells were used to lysate and perform western blot analysis. Data are presented as mean ± SD (n = 3; *, p < 0.05; ***, p < 0.005). **G-H** MCF7^WT-TRIP-Br1^ and MCF7^KD-TRIP-Br1^ cells were transfected with siNEDD4-1 and treated with MG132 (10 µM) for the indicated times, and the cells were collected for the western blot analysis. The quantification of the western blots is shown as the mean ± SD (n = 3; *; p < 0.05).

**Supplementary Figure 4.**

**
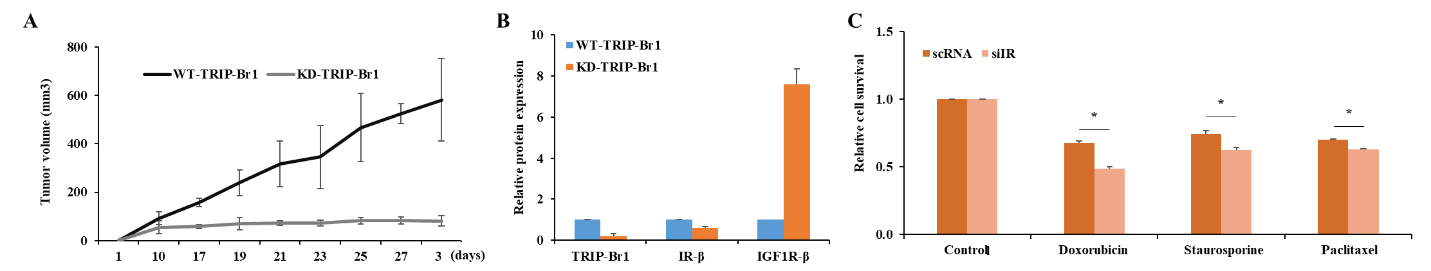
**

**Supplementary Figure 4. Enhanced tumor formation is associated with a higher IR/IGF1R ratio resulting from TRIP-Br1 expression. A-B** Nude mice were subcutaneously injected in the flanks with MCF7^WT-TRIP-Br1^ and MCF7^KD-TRIP-Br1^ cells. The tumor volume was measured at the indicated times and calculated as described in Materials and Methods. **C** MCF7 cells were transfected with IR silencing RNA for 24 h, seeded in 96-well plates, and treated with three different types of anticancer drugs, doxorubicin (1 µM), stauroseporine (0.1 µM) and paclitaxel (0.5 µM) for 24 h. The cell viability was measured as mentioned in the Materials and Methods section. Data are presented as the mean ± SD (n = 3; *, p < 0.05).

**Supplementary Figure 5.**

**
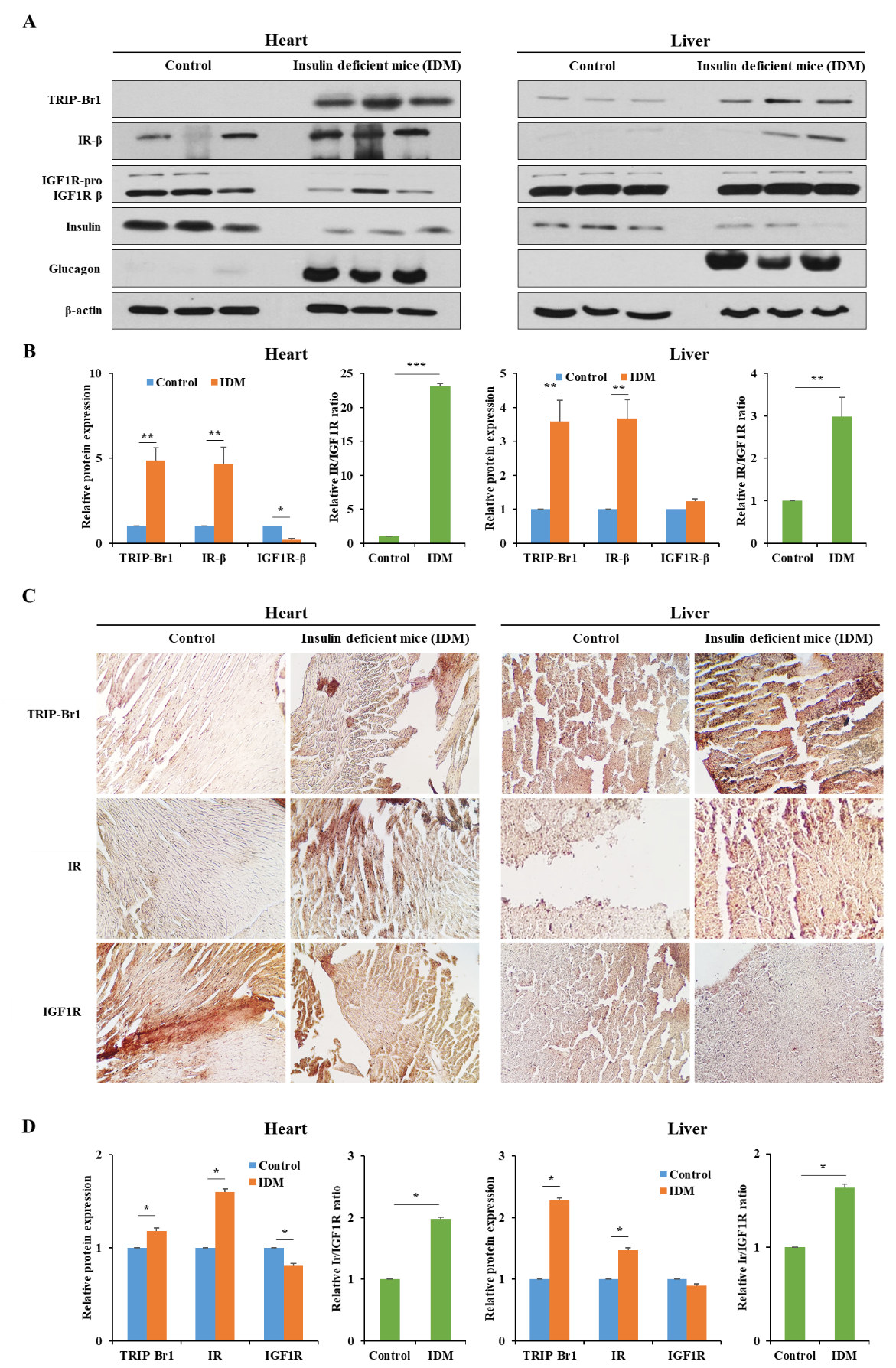
**

**Supplementary Figure 5. Supplementary results for the TRIP-Br1 mediated higher IR/IGF1R ratio in insulin-deficient mice mimicking** **diabetes. A-B** Tissue samples from the heart and liver were collected from 5-week-old insulin-producing mice (control) or insulin-deficient mice (IDM). The tissues were used to assess the levels of TRIP-Br1, IR, and IGF1R IR by western blot analysis, in which insulin and glucagon were used as controls. The quantification of results are presented as the mean ± SD (n > 3; *, p < 0.05; **, p < 0.01; ***, p < 0.005). The relative IR/IGF1R ratio is also shown. **C-D** Representative images of IHC analysis showing the expression levels of TRIP-Br1, IR, and IGF1R in the heart and liver of control or IDM groups. The expression levels of TRIP-Br1, IR, and IGF1R are presented as the mean ± SD (n > 3; *, p < 0.05; ***, p < 0.005). The relative IR/IGF1R ratio is also shown.

**Supplementary Table 1.** Patient characteristics base on single cell from 11 patients.


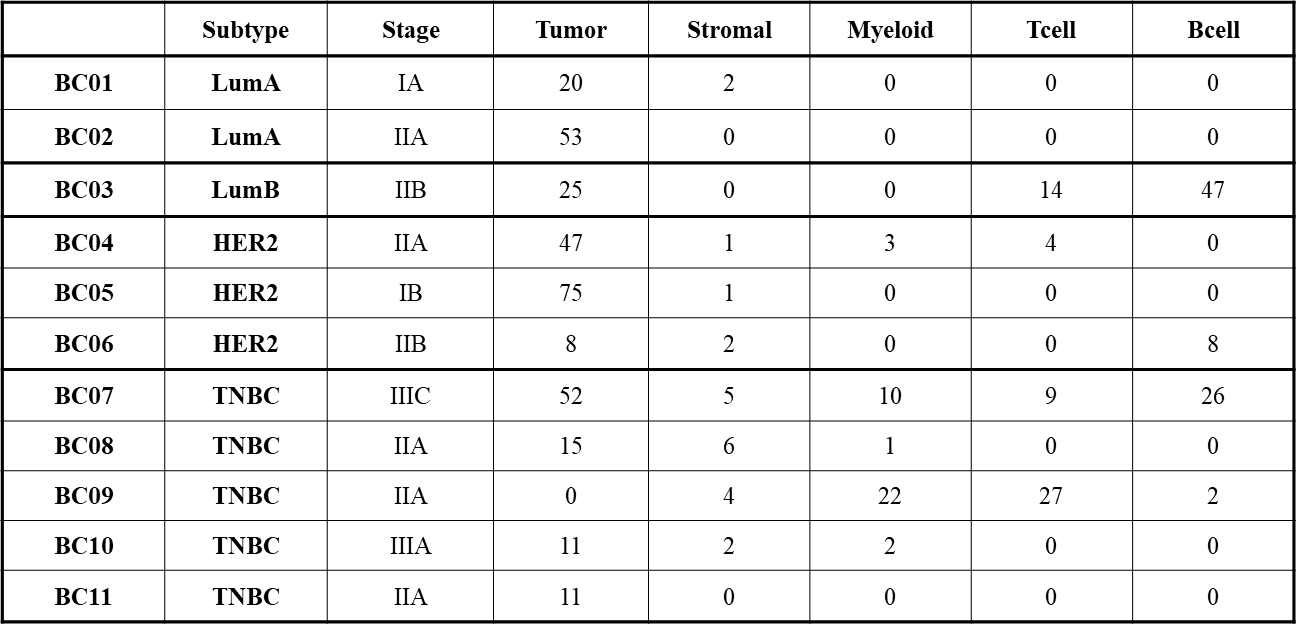


**Supplementary Figure 6.**

**
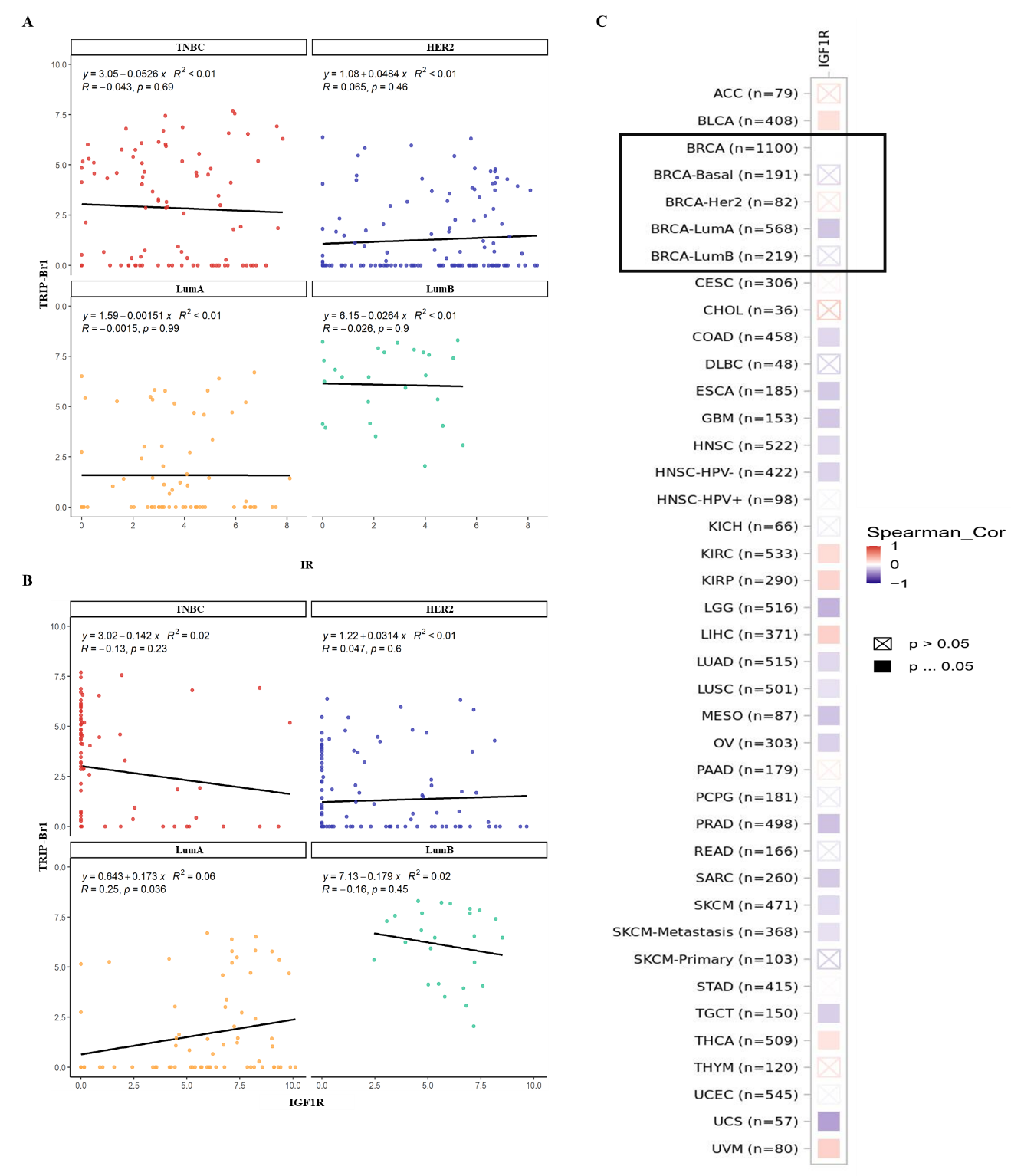
**

**Supplementary Figure 6. Supplementary results for the single cell analysis of the TRIP-Br1-mediated IR/IGF1R ratio. A** The relationship of TRIP-Br1 and IR expression in four different subtypes of breast cancer: TNBC, HER2, LumA, and LumB. **B** The relationship of TRIP-Br1 and IGF1R expression in four different subtypes of breast cancer: TNBC, HER2, LumA, and LumB. **C** Analysis of the relationship of TRIP-Br1 and IGF1R expression by using timer2.0 tool (<http://timer.cistrome.org/>).

**Supplementary Figure 7.**

**
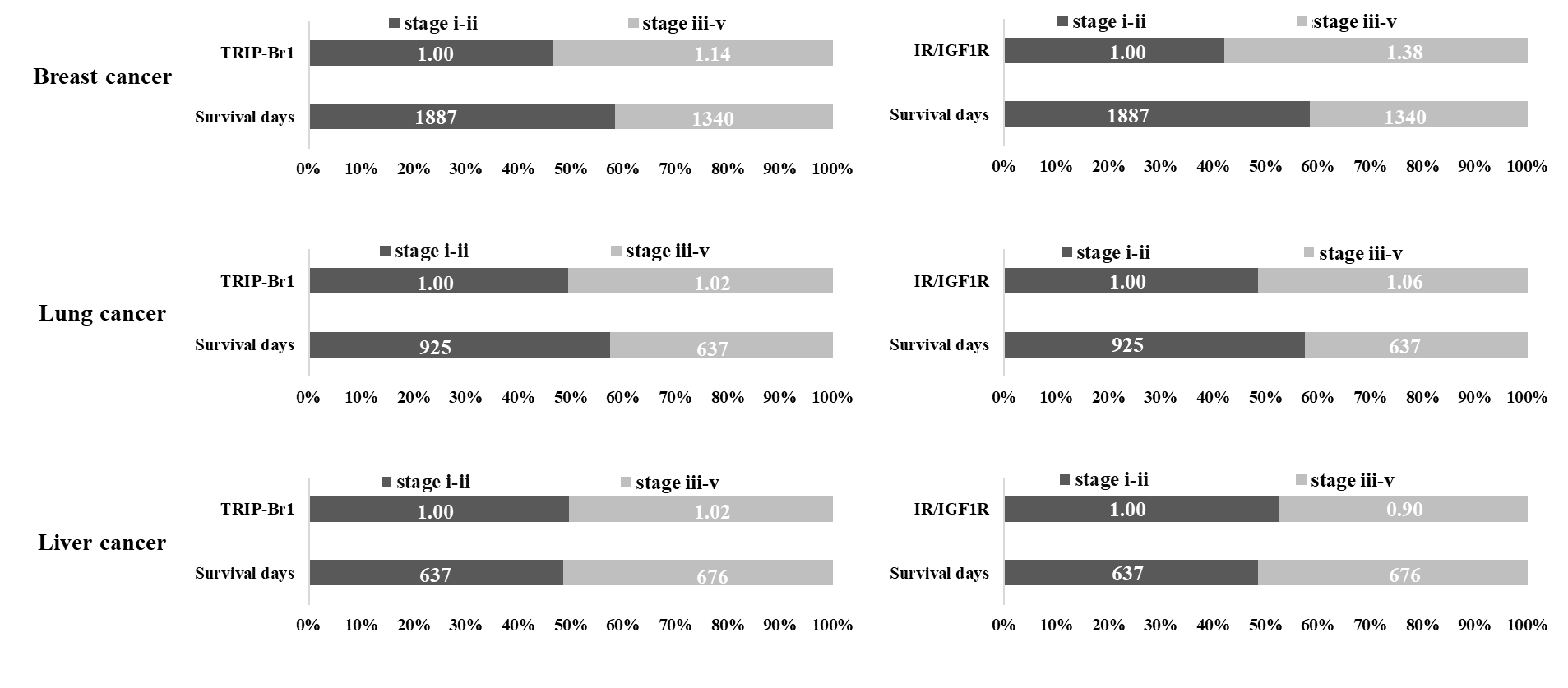
**

**Supplementary Figure 7. Bioinformatics analysis of TRIP-Br1-mediated IR/IGF1R ratio in other types of cancers** The relationship between survival days and TRIP-Br1 expression or the IR/IGF1R ratio was analyzed in two groups (stage i-ii and stage iii-v) of indicated cancer patients using the TCGA database.

**Supplementary Figure 8.**

**
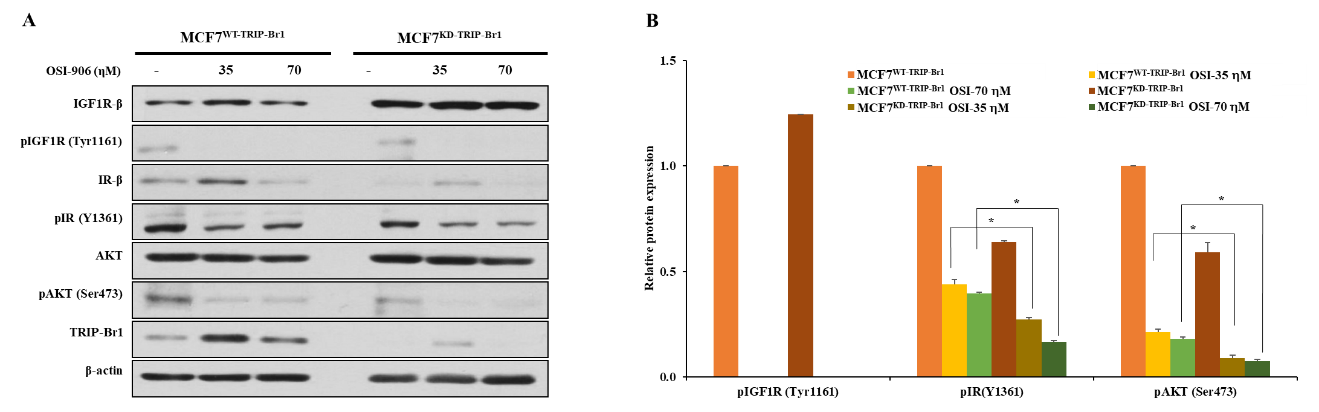
**

**Supplementary Figure 8. Higher IR/IGF1R ratio-mediated activation of the PI3K-AKT signaling pathway. A-B** MCF7^WT-TRIP-Br1^ and MCF7^KD-TRIP-Br1^cells were treated with OSI-906 for 24 h. The indicated protein levels were examined by western blot analysis. Data are presented as the mean ± SD (n > 3; *, p < 0.05).

**Supplementary references**

1. Pablo J Fernandez-Marcos 1, Cristina Pantoja, Agueda Gonzalez-Rodriguez, Nicholas Martin, Juana M Flores, Angela M Valverde, Eiji Hara, Manuel Serrano. Normal proliferation and tumorigenesis but impaired pancreatic function in mice lacking the cell cycle regulator sei1. PloS One. 2010;5(1):e8744.
