# Supplementary material for: The regulation of insulin receptor/insulin-like growth factor 1 receptor ratio, an important factor for breast cancer prognosis, by TRIP-Br1
Source: J Hematol Oncol. 2022 Jun 16;15:82. doi: 10.1186/s13045-022-01303-6 (PMC9204904; doi:10.1186/s13045-022-01303-6)
Supplement: Supplementary file 2 — Additional file 2: Supplementary Introduction and Results. [file 13045_2022_1303_MOESM2_ESM.docx]

**Introduction**

According to the World Health Organization (WHO), cancer remains a major threat to human health, and is currently the second most common cause of death [1]. Breast cancer is the most common type of cancer in women, with 19.3 million cases and 10 million cancer-related deaths reported in 2020 [2]. Thus, there is an urgent need to identify potential targets involved in breast cancer to aid in the development of efficient therapies. The relationship between breast cancer and diabetes has been extensively studied [3-8]. Women with diabetes are at a greater risk of developing breast cancer than those without diabetes [8-9]. This study attempts to understand the molecular mechanisms involved in both breast cancer and diabetes.

IR and IGF1R have been well studied in breast cancer and diabetes therapies. They share very high sequence homology and functional structures, such as the intracellular kinase domain [10-11]. In normal cells, both IR and IGF1R are activated by insulin and IGF1, which are well-known growth and survival factors. Insulin and IGF1 bind to IR and IGF1R in the form of homodimers or heterodimers with a very high affinity [12-14]. The binding of insulin and IGF1 to both receptors activates intrinsic receptor tyrosine kinase and downstream signaling cascades, which in turn regulate many cellular functions, including gene transcription, nutrient metabolism (glucose, lipids, and proteins), and cell growth and differentiation [15-17]. However, the aberrant expression and activation of IR and IGF1R are strongly associated with a greater risk of breast cancer [18-19]. IR and IGF1R are overexpressed in most cancer cells, including breast cancer cells [20-21]. Upregulated IR and IGF1R expression exacerbates tumorigenesis in cancer cells by activating many signaling pathways, including the phosphatidylinositol 3‑kinase (PI3K)/protein kinase B (AKT) and mitogen-activated protein kinase (MAPK) pathways. IR- and IGF1R-mediated signaling pathways are highly activated in more than 75% of breast cancer patients and 87% of invasive breast cancer patients [22]. High levels of IR and IGF1R signaling pathway activation is closely associated with mammary tumor growth and proliferation, angiogenesis, immune suppression, metastasis, invasion, and suppressed apoptosis, which can result in the development of aggressive breast cancer [23-26]. Therefore, IR and IGF1R have received considerable attention as prominent targets for cancer prevention and therapy [27-28]. However, many cancer treatment studies have focused on targeting the expression and activation of either IR or IGF1R without considering the effects of both IR and IGF1R. An approach that co-targets both receptors may enhance the antitumor efficacy of the treatment of cancers.

It has been suggested that the inhibition of IGF1R alone does not affect tumor growth in preclinical trials, but enhances the IR signaling pathway [29]. IR has also been reported to enhance multistage tumor progression and convey intrinsic resistance to IGF1R targeted therapy, as an essential part of the tumor-promoting IGF signaling pathway [30]. Interestingly, a recent study suggested that the IR/IGF1R ratio is a key factor in breast cancer prognosis [31]. Gallagher et al*.* evaluated the IR/IGF1R ratio in over 500 patients with breast cancer [31]. They showed that breast cancer patients with a higher IR/IGF1R ratio due to elevated IR expression not only have hyperinsulinemia but are also more susceptible to tumorigenesis-promoting effects due to a greater sensitivity to the growth-promoting effects of insulin [31]. In an attempt to determine how the IR/IGF1R ratio can be regulated in breast cancer cells, we initially focused on the TRIP-Br1 oncogenic protein.

Previously, we showed that a deficiency of insulin or IGF1 greatly increased TRIP-Br1 gene expression in breast cancer cells. However, this expression was decreased to basal levels after the addition of insulin or IGF1, implying that TRIP-Br1 might be associated with insulin and IGF1-related signaling pathways [32]. Interestingly, TRIP-Br1 was upregulated genes in type 1and type 2 diabetes [33]. Fernandez-Marcos et al. concluded that Sei1 (=TRIP-Br1) plays an important role in diabetes or metabolic disorders [34]. In addition, TRIP-Br1 was found to be as one of 11 up-regulated gene in both type 1 and type 2 diabetes when the gene expression in pancreatic alpha cells from type 1 and type 2 diabetes was analyzed [33]. Our own research and other groups have shown that TRIP-Br1 is significantly overexpressed in various cancers [32]. We also found that TRIP-Br1 expression greatly increased in various cancer cell lines, especially in breast cancer cell lines, compared to other types of cancer cell lines, in response to cell death-inducing stressful conditions (e.g., nutrient starvation and anticancer treatment) [32]. Upregulated TRIP-Br1 suppresses programmed cell death, such as apoptosis and necroptosis, in breast cancer cells as an oncoprotein [32]. TRIP-Br1 is known to be involved in various biological functions, including transcription, cell cycle progression, metabolism, programmed cell death, metastasis, invasion, and tumorigenesis [35-37]. TRIP-Br1 contains multiple protein-interacting domains, including an N-terminal putative cyclin-A-binding domain, a novel highly conserved SERTA domain, a binding motif for PHD zinc finger- and/or bromodomain-containing proteins, and an acidic C-terminal domain. These various protein-interacting domains seem to enable TRIP-Br1 to act as an adaptor protein [38-41]. For example, TRIP-Br1 directly binds to two E3 ubiquitin ligases (NEDD4-1 and XIAP) and their target proteins (PTEN and adenylyl cyclase) as an adaptor protein, in which TRIP-Br1/E3 ligases induce the ubiquitination and degradation or cellular translocation of the target proteins [39-41].

In this study, the regulatory mechanism of TRIP-Br1 in controlling the IR/IGF1R ratio in breast cancer cells is explored.

**Supplementary Results**

**Result S1. Supplementary results for the positive impact of TRIP-Br1 on IR expression**

The effects of TRIP-Br1 expression on the IR/IGF1R ratio were initially evaluated in normal and breast cancer cell lines. IR and IGF1R expression levels were normalized in MCF10A to compare the IR/IGFR1 ratio in each breast cancer cell line. The majority of the breast cancer cell lines showed significantly higher levels of IR expression than IGF1R expression, resulting in a high IR/IGF1R ratio **(Figure 1A-B)**. In particular, four cancer cell lines (MDA-MB-453, MDA-MB-468, BT20, and BT549) with very high levels of TRIP-Br1 showed a much higher IR/IGF1R ratio than the other cancer cell lines **(Figure 1A-B)**. In our previous and unpublished studies, we found that TRIP-Br1 gene expression was always very high regardless of stressful stimuli in these four cell lines, while TRIP-Br1 expression was greatly increased in MCF7 and MDA-MB-231 cell lines in response to various cell death-inducing stressful conditions [32]. Therefore, the MCF7 and MDA-MB-231 cell lines were selected for further study.

First, the impact of TRIP-Br1 on IR expression was tested in MEFs isolated from TRIP-Br1 wild-type (MEF^WT-TRIP-Br1^) and knockout (MEF^KO-TRIP-Br1^) mice. Confocal immunofluorescence experiment revealed a higher level of IR expression in MEF^WT-TRIP-Br1^ compared to MEF^KO-TRIP-Br1^ cells (**Figure S1A-B**). The knockout of TRIP-Br1 was confirmed by genotyping, as shown in **Figure S1C and S1F**. Interestingly, we found an inverse relationship between IR and IGF1R expression. IR silencing significantly increased the IGF1R protein levels in MCF7 and MDA-MB-231 cells **(Figure S1I-J).**

Taken together, these data clearly indicate that TRIP-Br1 positively regulates the expression of IR at the protein level, thereby enhancing the IR/IGF1R ratio.

**Result S2. Supplementary results for the negative effect of TRIP-Br1 on IGF1R expression**

TRIP-Br1 was found to contribute to a relatively high IR/IGF1R ratio by positively affecting IR expression. In addition, we examined the effects of TRIP-Br1 on IGF1R expression. First, the effect of TRIP-Br1 on IGF1R expression was tested in MCF7 and MDA-MB-231 cells. While TRIP-Br1 overexpression significantly decreased IGF1R expression **(Figure S2A-B**), TRIP-Br1 silencing greatly increased IGF1R expression (**Figure S2C-D**). In addition, MCF7^KD-TRIP-Br1^ cells also showed much higher IGF1R expression than MCF7^WT-TRIP-Br1^ cells **(Figure S2E-F**). Lastly, TRIP-Br1 knockout mice showed elevated IGF1R in the heart (~ 2-fold) compared to control mice (**Figure S2G-H**). As TRIP-Br1 is highly expressed in adipocytes, and because TRIP-Br1 directly regulates and facilitates IGF1R degradation but not IR, thus IR seems to be less affected by TRIP-Br1-KO in adipocytes.

Overall, these data strongly suggest that TRIP-Br1 negatively affects IGF1R expression, eventually increasing the IR/IGF1R ratio in breast cancer cells.

**Result S3. Supplementary results for the TRIP-Br1/NEDD4-1 mediated IGF1R degradation**

Next, we investigated how TRIP-Br1 downregulates the IGF1R protein levels. TRIP-Br1 directly binds to two E3 ubiquitin ligases, NEDD4-1 and XIAP, as an adaptor protein [40,41]. In addition, multiple lines of evidence have indicated that NEDD4-1 is an E3 ubiquitin ligase responsible for IGF1R degradation [42-46]. For example, oxidative stress-mediated NEDD4-1 upregulation degrades IGF1R during neurodegeneration [42]. However, no direct interaction between the IGF1R and NEDD4-1 has been reported, implying a possible constraint, such as the need for an adaptor protein. Therefore, we investigated whether TRIP-Br1 is responsible for the ubiquitination and degradation of IGF1R by interacting with NEDD4-1 or XIAP. Interestingly, the IGF1R expression levels were greatly increased in TRIP-Br1 and/or NEDD4-1 silenced cells. However, little change was observed in TRIP-Br1/XIAP double knockdown cells **(Figure S3A-B**). The effect of NEDD4-1 on IGF1R degradation was also assessed. The IGF1R protein levels increased considerably after NEDD4-1 silencing in the presence of cycloheximide (CHX), a protein synthesis blocker (**Figure S3C-D**).

The degradation of many ligand-induced receptors is mediated through the ubiquitination of the receptors, followed by proteasome- or lysosome-dependent degradation. The binding of IGF1 to IGF1R leads to the polyubiquitination of IGF1R [47]. Our previous and unpublished data showed that TRIP-Br1 plays an important role in both pathways [40]. Thus, we evaluated which pathway is responsible for TRIP-Br1/NEDD4-1 mediated IGF1R degradation. To test this hypothesis, siNEDD4-1 was transfected into MCF7^WT-TRIP-Br1^ and MCF7^KD-TRIP-Br1^ cells in the absence or presence of MG132 or CQ. NEDD4-1 silencing significantly increased the IGF1R protein levels in the presence of MG132 but only slightly increased after CQ treatment in MCF7^WT-TRIP-Br1^ cells **(Figure S3E-F)**. Similar results were obtained for the IGF1R expression levels after treatment with MG132 **(Figure S3G-H**). These findings suggest that TRIP-Br1/NEDD4-1-mediated IGF1R degradation occurs mainly through the proteasome/ubiquitination pathway rather than through a lysosomal pathway.

**Result S4. Enhanced tumor formation is associated with a higher IR/IGF1R ratio resulting from TRIP-Br1 expression**

Next, we examined the IR/IGF1R ratio in TRIP-Br1-mediated tumor formation and growth using a xenograft model. MCF7^WT-TRIP-Br1^ and MCF7^KD-TRIP-Br1^ cells were subcutaneously injected into nude mice and tumor size was measured on the indicated days (**Figure S4A**). Our results revealed a significant reduction in the tumor volume in MCF7^KD-TRIP-Br1^ pretreated mice, suggesting that TRIP-Br1 is effective in strengthening *in vivo* tumor formation and growth ([**Figure**](https://www.nature.com/articles/cddis2017272#Fig6) **S4A**). In agreement with the *in vitro* observations, an approximately 10-fold higher IR/IGF1R ratio, due to the higher IR but lower IGF1R, was detected in MCF7^WT-TRIP-Br1^ cells grown in null mice ([**Figure**](https://www.nature.com/articles/cddis2017272#Fig6) **S4B**). This suggests that a higher IR/IGF1R ratio could enhance the growth and proliferation of breast cancer cells. This result is consistent with those of previous studies. Although the inhibition of IGF1R was previously reported to not affect tumor growth in preclinical trials, it was found to enhance the IR signaling pathway, enhancing multistage tumors as a result [29-30]. These results suggest that IR, rather than IGF1R, may be responsible for the better growth and survival of cancer cells. This hypothesis was tested by examining the effect of IR on the survival of MCF7 cells in response to three different anticancer drugs (doxorubicin, staurosporine, and paclitaxel), namely, resistance to anticancer-mediated cell death. The MCF7 cell line is well known for its high resistance to programmed cell death against various anticancer drugs. Cell viability was found to be lower in IR-silenced MCF7 cells than in control cells after treatment with anticancer drugs (**Figure S4C).**

Altogether, these results indicate that TRIP-Br1 provides breast cancer cells with a better capacity for proliferation and survival by increasing the IR/IGF1R ratio even after treatment with anticancer drugs.

**Result S5. Supplementary results for the TRIP-Br1 mediated higher IR/IGF1R ratio in insulin-deficient mice mimicking** **diabetes**

The effect of TRIP-Br1 on the IR/IGF1R ratio and the inverse relationship between IR and IGF1R expression were further tested in insulin-deficient mice mimicking diabetes patients, in which decreased insulin and increased glucagon levels were used as controls (**Figure S5A-B)**. As in our previous studies, in which a deficiency of insulin or IGF1 was found to trigger TRIP-Br1 upregulation in breast cancer cells [32], a similar pattern was observed in our animal model (**Figure S5A-B)**. Insulin-deficient mice showed significantly elevated TRIP-Br1 protein levels, which were accompanied by increased IR but decreased IGF1R in heart and liver tissue samples, confirming the positive effect of TRIP-Br1 on the higher IR/IGF1R ratio (**Figure S5A-B)**. Representative images of immunohistochemical staining for TRIP-Br1, IR, and IGF1R in insulin-deficient mice and the corresponding normal mouse tissues are shown in **Figure S5C**. Again, significantly higher TRIP-Br1 and IR, but lower IGF1R expression levels, were observed in insulin-deficient mice compared to normal mouse tissues, resulting in a higher IR/IGF1R ratio in insulin-deficient mice **(Figure S5D)**. These findings strongly suggest that TRIP-Br1 also positively regulates IR but negatively regulates IGF1R expression, resulting in a higher IR/IGF1R ratio, in insulin-deficient mice.

Taken together, these results imply that TRIP-Br1 is also at least partly responsible for the induction of a higher IR/IGF1R ratio in patients with diabetes as well as breast cancer.

**Result Table 1 and S6. Supplementary results for the TRIP-Br1-mediated IR/IGF1R ratio**

To further elucidate the relationship between TRIP-Br1 expression and the IR/IGF1R ratio, we analyzed 317 tumor single cells from 11 breast cancer patients, as shown in GSE75688 datasets, which are divided into four representative subtypes **(Table 1)**. Unexpectedly, no significant relationship was found between TRIP-Br1 and IR expression in any of the four subtypes **(Figure S6)**. However, triple-negative breast cancer (TNBC, HR^-^/HER2^-^) tumor cells showed a negative correlation between TRIP-Br1 and IGF1R expression **(Figure S6B)**. Accordingly, a positive correlation between the TRIP-Br1 expression levels and the IR/IGF1R ratio was found in TNBC. The TNBC subtype is known to induce the lowest survival rate in breast cancer patients compared to other subtypes. In addition, a study from the same GSE75688 datasets revealed that TNBC showed higher EMT and recurrence scores than luminal subtypes [48]. These results imply that TNBC with a TRIP-Br1-mediated higher IR/IGF1R ratio may lead to worse tumor progression and metastasis. The luminal A subtype (LumA, HR^+^/HER2^-^) showed the opposite results, in which only two patients were tested. However, our bioinformatics analysis (<http://timer.cistrome.org/>) from the database, with as many as 568 patients, showed an inverse relationship between TRIP-Br1 and IGF1R expression, similar to our *in vitro* results **(Figure S6C).** Interestingly, an inverse relationship between IR and IGF1R expression was observed in these two subtypes. These data confirmed a strong relationship between TRIP-Br1 and IR/IGF1R ratio with tumor progression and metastasis in breast cancer patient.

**Result S7. Bioinformatics analysis of TRIP-Br1-mediated IR/IGF1R ratio in other types of cancers**

In the present study, TRIP-Br1-mediated higher IR/IGF1R ratio was found to enable the proliferation and survival of breast cancer cells. In addition to our invaluable study on the importance of the IR/IGF1R ratio in breast cancer research, it would be very interesting to extend our research to other types of cancers. Therefore, we evaluated the effect of the TRIP-Br1-mediated IR/IGF1R ratio on the survival time of patients with other types of cancer, in addition to breast cancer patients, using the Cancer Genome Atlas (TCGA) database. Based on the mRNA levels of TRIP-Br1, IGF1R, and IR from TCGA database, we determined the survival time of three types of cancer patients at two different stages (stage i-ii and iii-x). Our bioinformatics analysis revealed that TRIP-Br1 was positively correlated with the IR/IGF1R ratio but inversely correlated with survival time in breast cancer patients (n = 152). However, no significant relationship was observed with lung (n = 396) or liver cancer (n = 130) (**Figure S7**). This implies that TRIP-Br1 may be a breast cancer-specific oncogenic adaptor protein.

**Result S8. Higher IR/IGF1R ratio-mediated activation of the PI3K-AKT signaling pathway**

We showed that IR negatively affected IGF1R expression. In contrast, we also attempted to elucidate the effect of the IGF1R on IR expression. Although the exact mechanism by which IGF1R regulates IR remains to be fully understood, it has been proposed that IGF1R downregulation does not directly affect IR expression, but rather increases sensitivity to insulin [49]. IR and IGF1R can activate the PI3K-AKT signaling pathway, which enhances the proliferation and survival of cancer cells. Our findings also indicated that the phosphorylation level of AKT on the Ser473 residue was markedly reduced when treated with OSI-906, a dual inhibitor of IGF1R and IR **(Figure S8A-B),** suggesting that IGF1R and IR positively regulate the AKT signaling pathway. Zhang et al*.* showed that the insulin-mediated phosphorylation of Akt was greatly enhanced when IGF1R was downregulated in breast cancer cells [49]. They proposed that the increase in insulin signaling upon IGF1R downregulation is a common phenomenon among breast cancer cells [49]. Taking our findings together, we propose the hypothesis that a higher IR/IGF1R ratio, with upregulated IR but downregulated IGF1R, may highly activate the PI3K-AKT signaling pathway, which subsequently enhances the proliferation and survival of cancer cells.

In summary, TRIP-Br1 does not directly interact with IR but suppresses the proteasome-mediated degradation ubiquitination and degradation of IR, implying the negative effect on unknown E3 ligase, most likely CHIP or MARCH1. By contrast, TRIP-Br1 directly interacts with both IGF1R and NEDD4-1 E3 ubiquitin ligase, in which TRIP-Br1/NEDD4-1 degrades IGF1R via the ubiquitin/proteasome system rather than the lysosomal pathway. Eventually TRIP-Br1 increased the IR/IGF1R ratio and most likely worsened the prognosis of breast cancer patients. In addition, downregulated IGF1R is known to induce the IR activation, which eventually activates the PI3K/AKT and MAPK signaling pathways, resulting in the cancer cell proliferation and survival (**Figure 2J**)

In conclusion, our findings provide valuable information on the regulatory mechanisms of the IR/IGF1R ratio. In this study, we showed that a TRIP-Br1-mediated higher IR/IGF1R ratio increased the survival rate of breast cancer cells, resulting in a worse prognosis for cancer patients. Therefore, the TRIP-Br1-mediated IR/IGF1R ratio appears to be a predictive factor for the prognosis and progression of cancer.

**Supplementary references**

1. Wang H, Naghiavi M, Allen C, Barber R, Bhutta Z, Carter AR, et al. Global, regional, and national life expectancy, all-cause mortality, and cause-specific mortality for 249 causes of death. The Lancet. 2016;388(10053):1459-1544.

2. Sung H, Ferlay J, Siegel RL, Laversanne M, Soerjomataram I, Jemal A, et al. Cancer statistics 2020: GLOBOCAN estimates of incidence and mortality worldwide for 36 cancers in 185 countries. CA Cancer J Clin. 2021;71:209-249.

3. Garg SK, Maurer H, Reed K, Selagamsetty R. Diabetes and cancer: two diseases with obesity as a common risk factor. Diabetes Obes Metab. 2014;16(2):97-110.

4. Giovannucci E, Harlan DM, Archer MC, Bergenstal RM, Gapstur SM, Habel LA, et al. Diabetes and cancer: a consensus report. Diabetes Care. 2010; 33(7):1674-1685.

5. Maskarinec G, Jacobs S, Park SY, Haiman CA, Setiawan VW, Wilkens LR, et al. Type II Diabetes, Obesity, and Breast Cancer Risk: The Multiethnic Cohort. Cancer Epidemiol Biomarkers Prev. 2017; 26(6):854-861.

6. Larsson SC, Mantzoros CS, Wolk A. Diabetes mellitus and risk of breast cancer: A meta‐analysis. Int J Cancer. 2007;121:856–862.

7. Wolf I, Sadetzki S, Catane R, Karasik A, Kaufman B. Diabetes mellitus and breast cancer. Lanet Oncol. 2005;6(2):103-111.

8. Martin SD, McGee SL. Metabolic reprogramming in type 2 diabetes and the development of breast cancer. J Endocrinol. 2018;237(2):35-46.

9. Bronsveld HK, Jensen V, Vahl P, De Bruin ML, Cornelissen S, Sanders J, et al. Diabetes and Breast Cancer Subtypes. PloS One. 2017; 12(1):e0170084.

10. Cai W, Sakaguchi M, Kleinridders A, Pino GGD, Dreyfuss JM, O’Neill BT, et al. Domain-dependent effects of insulin and IGF-1 receptors on signalling and gene expression. Nat Commun. 2017;8:14892.

11. Ullrich A, Gray A, Tam AW, Yang-Feng T, Tsubokawa M, Collins C, et al. Insulin like growth factor I receptor primary structure comparison with insulin receptor suggests structural determinants that define functional specificity. The EMBO J. 1986;5(10):2503-2512.

12. Swinnen SG, Hoekstra JB, DeVries JH. Insulin therapy for type 2 diabetes. Diabetes Care. 2009;32(Suppl 2):S253-9.

13. Teppala S, Shankar A. Association between serum IGF-1 and diabetes among U.S. adults. Diabetes Care. 2010;33(10):2257-2259.

14. Cabail MZ, Li S, Lemmon E, Bowen M, Hubbard SR, Miller WT. The insulin and IGF1 receptor kinase domains are functional dimers in the activated state. Nat Commun. 2015;6:6406.

15. Soos MA, Whittaker J, Lammers R, Ullrich RA, Siddle K. Receptors for insulin and insulin-like growth factor-I can form hybrid dimers. Characterisation of hybrid receptors in transfected cells. Biochem J. 1990;270(2):383–390.

16. Belfiore A, Frasca F, Pandini G, Sciacca L, Vigneri R. Insulin receptor isoforms and insulin receptor/insulin-like growth factor receptor hybrids in physiology and disease. Endocr Rev. 2009;30(6):586-623.

17. Boucher J, Kleinridders A, Kahn CR. Insulin receptor signaling in normal and insulin-resistant states. Cold Spring Harb Perspect Biol. 2014;6(1):a009191.

18. Murphy N, Knuppel A, Papadimitriou N, Martin RM, Tsilidis KK, Smith-Byrne K, et al. Insulin-like growth factor-1, insulin-like growth factor-binding protein-3, and breast cancer risk: observational and Mendelian randomization analyses with approximately 430 000 women. Ann Oncol. 2020;31(5):641-649.

19. Eliassen AH, Tworoger SS, Mantzoros CS, Pollak MN, Hankinson SE. Circulating insulin and c-peptide levels and risk of breast cancer among predominately premenopausal women. Cancer Epidemiol Biomarkers Prev. 2007;16(1):161-164.

20. Girnita L, Takahashi SI, Crudden C, Fukushima T, Worrall C, Furuta H, et al. Chapter Seven - When Phosphorylation Encounters Ubiquitination: A Balanced Perspective on IGF-1R Signaling. Prog Mol Biol Transl Sci. 2016;141:277-311.

21. Brahmkhatri VP, Prasanna C, Atreya HS. Insulin-like growth factor system in cancer: novel targeted therapies. Biomed Res Int. 2015;2015:538019.

22. Ireland L, Santos A, Campbell F, Figueiredo C, Hammond D, Ellies LG, et al. Blockade of insulin-like growth factors increases efficacy of paclitaxel in metastatic breast cancer. Oncogene. 2018;37(15):2022-2036.

23. Rostoker R, Abelson S, Bitton-Worms K,Genkin I, Ben-Shmuel S, Dakwar M, et al. Highly specific role of the insulin receptor in breast cancer progression. Endocr Relat Cancer. 2015;22(2):145-157.

24. Pollak M. The insulin and insulin-like growth factor receptor family in neoplasia: an update. Nat Rev Cancer. 2012;12(3):159-169.

25. Hua H, Kong Q, Yin J, Jiang Y. Insulin-like growth factor receptor signaling in tumorigenesis and drug resistance: a challenge for cancer therapy. J Hematol Oncol. 2020;13(1):64.

26. Malaguarnera R, Belfiore A. The Insulin Receptor: A New Target for Cancer Therapy. Front Endocrinol (Lausanne). 2011;2:93

27. Sun Y, Sun X, Shen B. Molecular Imaging of IGF-1R in Cancer. Mol Imaging. 2017;16:1536012117736648.

28. Pian L, Wen X, Kang L, Li Z, Nie Y, Du Z, et al. Targeting the IGF1R Pathway in Breast Cancer Using Antisense lncRNA-Mediated Promoter cis Competition. Mol Ther Nucleic Acids. 2018;12:105-117.

29. Buck E, Gokhale PC, Koujak S, Brown E, Eyzaguirre A, Tao N, et al. Compensatory insulin receptor (IR) activation on inhibition of insulin-like growth factor-1 receptor (IGF-1R): rationale for cotargeting IGF-1R and IR in cancer. Mol Cancer Ther. 2010;9(10):2652-64.

30. Ulanet DB, Ludwig DL, Kahn CR, Hanahan D. Insulin receptor functionally enhances multistage tumor progression and conveys intrinsic resistance to IGF-1R targeted therapy. PNAS. 2010;107(24):10791-10798.

31. Gallagher EJ, Fei K, Feldman SM, Port E, Friedman NB, Boolbol SK, et al. Insulin resistance contributes to racial disparities in breast cancer prognosis in US women. Breast Cancer Res. 2020;22(1):40.

32. Jung S, Li C, Duan J, Lee S, Kim K, Park Y, et al. TRIP-Br1 oncoprotein inhibits autophagy, apoptosis, and necroptosis under nutrient/serum-deprived condition. Oncotarget. 2015;6(30):29060-29075.

33. Bosi E, Marchetti P, Rutter GA, Eizirik DL. The gene signatures of human alpha cells in types 1 and 2 diabetes indicate disease-specific pathways of alpha cell dysfunction. Preprint at https://www.biorxiv.org/content/10.1101/2022.02.22.481528v1 (2022).

34. Fernandez-Marcos PJ, Pantoja C, Gonzalez-Rodriguez A, Martin N, Flores JM, Valverde AM, et al. Normal proliferation and tumorigenesis but impaired pancreatic function in mice lacking the cell cycle regulator sei1. PloS One. 2010;5(1):e8744.

35. Hong SW, Kim CJ, Park WS, Shin JS, Lee SD, Ko SG, et al. p34SEI-1 inhibits apoptosis through the stabilization of the X-linked inhibitor of apoptosis protein: p34SEI-1 as a novel target for anti-breast cancer strategies. Cancer Res. 2009;69(3):741-746

36. Hong SW, Shin JS, Lee YM, Kim DG, Lee SY, Yoon DH, et al. p34 (SEI-1) inhibits ROS-induced cell death through suppression of ASK1. Cancer Biol Ther. 2011;12(5):421-426.

37. Lai IL, Wang SY, Yao YL, Yang WM. Transcriptional and subcellular regulation of the TRIP-Br family. Gene. 2007;388(1-2):102-109.

38. Li J, Muscarella P, Joo SH, Knobloch TJ, Melvin WS, Weghorst CM, Tsai MD. Dissection of CDK4-binding and transactivation activities of p34(SEI-1) and comparison between functions of p34(SEI-1) and p16(INK4A). Biochemistry. 2015; 44(40):13246-13256.

39. Hu W, Yu X, Liu Z, Sun Y, Chen X, Yang X, et al. The complex of TRIP-Br1 and XIAP ubiquitinates and degrades multiple adenylyl cyclase isoforms. Elife. 2017;6:e28021.

40. Jung S, L4i C, Jeong D, Lee S, Ohk J, Park M, et al. Oncogenic function of p34SEI-1 via NEDD41mediated PTEN ubiquitination/degradation and activation of the PI3K/AKT pathway. Int J Oncol. 2013; 43(5):1587-1595.

41. Hong SW, Moon JH, Kim JS, Shin JS, Jung KA, Lee WK, et al. p34 is a novel regulator of the oncogenic behavior of NEDD4-1 and PTEN. Cell Death Differ. 2014;21(1):146-160.

42. Kwak YD, Wang B, Li JJ, Wang R, Deng Q, Diao S, et al. Upregulation of the E3 ligase NEDD4-1 by oxidative stress degrades IGF-1 receptor protein in neurodegeneration. J Neurosci. 2012;32(32):10971-10981.

43. Monami G, Emiliozzi V, Morrione A. Grb10/Nedd4-mediated multiubiquitination of the insulin-like growth factor receptor regulates receptor internalization. J Cell Physiol. 2008;216(2):426-437.

44. Huang Q, Szebenyi DM. Structural basis for the interaction between the growth factor-binding protein GRB10 and the E3 ubiquitin ligase NEDD4. J Biol Chem. 2010:285(53):42130-9.

45. Zhang Y, Goodfellow R, Li Y, Yang S, Winters CJ, Thiel KW, et al. NEDD4 ubiquitin ligase is a putative oncogene in endometrial cancer that activates IGF-1R/PI3K/Akt signaling. Gynecol Oncol. 2015;139(1):127-133.

46. Yan C, Zhao M, Li S, Liu T, Xu C, Liu L, et al. Increase of E3 ubiquitin ligase NEDD4 expression leads to degradation of its target proteins PTEN/IGF1R during the formation of goose fatty liver. J Ani Sci. 2020;98(9):skaa270.

47. Kavran JM, McCabe JM, Byrne PO, Connacher MK, Wang Z, Ramek A, et al. How IGF-1 activates its receptor. Elife. 2014;3:e03772.

48. Chung WS, Eum HH, Lee HO, Lee KM, Lee HB, Kim KT, et al. Single-cell RNA-seq enables comprehensive tumour and immune cell profiling in primary breast cancer. Nat Commun. 2017;8(1):15081.

49. Zhang H, Pelzer AM, Kiang DT, Yee D. Down-regulation of Type I Insulin-like Growth Factor Receptor Increases Sensitivity of Breast Cancer Cells to Insulin. Cancer Res. 2007;67(1):391-397.
