# Supplementary material for: The regulation of insulin receptor/insulin-like growth factor 1 receptor ratio, an important factor for breast cancer prognosis, by TRIP-Br1
Source: J Hematol Oncol. 2022 Jun 16;15:82. doi: 10.1186/s13045-022-01303-6 (PMC9204904; doi:10.1186/s13045-022-01303-6)
Supplement: Supplementary file 3 — Additional file 3: Materials and Methods. [file 13045_2022_1303_MOESM3_ESM.docx]

**Materials and Methods**

**Cell lines and reagents.** Cell lines were obtained from ATCC. Human MCF10A normal mammary epithelial cells were cultured as previously described [1]. Breast cancer cell lines and mouse embryonic fibroblast (MEF) cells were cultured in Dulbecco’s modiﬁed Eagle’s medium (DMEM) (Cat#31966–021; Life Technologies) supplemented with 10% fetal bovine serum (FBS) (Gibco) and a 1% antibiotic-antimycotic solution (Cat#15240-06, Gibco). All the cells were maintained at 37°C in a humidified atmosphere with 95% air and 5% CO_2_. The reagents were purchased from the following manufacturers: MG132 (Cat#M-1157; A.G. Scientific Inc.), chloroquine (CQ) (Cat#C6628; Sigma-Aldrich), cycloheximide (CHX) (Cat#C7698; Sigma), and OSI-906 (Cat#S1091; Selleckchem).

**Suppression or overexpression of TRIP-Br1, NEDD4-1, and IR.** To repress TRIP-Br1 expression, cells were transfected with TRIP-Br1 silencing siRNA (siTRIP-Br1) (Cat#sc-62988; Santa Cruz Biotechnology) using Lipofectamine 2000 (Cat#11668; Invitrogen) in Opti-MEM (Cat#31985; Invitrogen), in which scrambled small interfering RNA (scRNA) was used as a control. MCF7^WT-TRIP-Br1^ and MCF7^KD-TRIP-Br1^ stable cell lines were established as described in our previous study [2]. To establish TRIP-Br1 overexpressing cells, cells were transfected with TRIP-Br1 overexpressing plasmid (pcDNA3.1/TRIP-Br1) using Lipofectamine 2000 in Opti-MEM medium. NEDD4-1 and IR genes were silenced using TurboFect Transfection Reagent (Cat #R0531; Thermo Scientific) with NEDD4-1 silencing siRNA (siNEDD4-1) or IR silencing RNA (siIR) from BIONEER Corporation (Korea) as follows:

siNEDD4-1: UUCCAUGAAUCUAGAAGAACATT/UGUUCUUCUAGAUUCAUGGAATT

siIR: GCAGGUCCCUUGGCGAUGU/ACAAGACCUAAGUGCACUG=tt

**Cell viability analysis.** Cell viability was analyzed using the Cell Viability, Proliferation & Cytotoxicity Assay Kit (Cat#EZ-3000; EZ-CYTOX) in accordance with the manufacturer’s instructions by measuring the absorbance at 450 nm using a Gemini XPA Microplate Reader.

**Western blot analysis.** Western blot analysis of cell lines was performed as previously described [3]. For the western blot analysis of mouse tissue samples, tissues from mice were dissected, washed with PBS, and homogenized in RIPA buffer (50 mM Tris-HCl, 150 mM NaCl, 0.1% SDS, 0.5% sodium deoxycholate, 1% TritonX-100, 1 mM DTT, 1 mM PMSF, pH 7.4) supplemented with a protease inhibitor cocktail (Cat#P-1512; A.G. Scientific Inc.). After sonication, the tissue lysates were centrifuged at 16,000 × *g* for 20 min, and the supernatants were used for western blotting. The following antibodies were used: TRIP-Br1 (Cat #ALX -804-645; Enzo Life Sciences), IR (Cat#57982; Cell Signaling), IGF1R (Cat#sc-81464; Santa Cruz Biotechnology, or Cat#ab39675; Abcam), IGF1R/IR (Cat#ab172965; Abcam), NEDD4-1 (Cat#sc-25508; Santa Cruz Biotechnology), insulin (Cat#ab63820; Abcam), glucagon (Cat#ab92517; Abcam), and β-actin (Cat#sc-47778; Santa Cruz Biotechnology).

**Quantitative real time polymerase chain reaction (qRT-PCR).** qRT-PCR was performed by employing LightCycler 96 detection system (Roche). Total RNA was extracted from MCF7 cells using GeneAll® RiboEx™ (GeneAll; Cat#301-001) according to manufacturer’s protocol. Complementary DNAs (cDNAs) were synthesized through the reverse transcription of 1µg of RNA using AccuPower® CycleScript RT PreMix (Bioneer; Cat#K-2044) according to manufacturer’s protocol. qRT-PCR reactions were performed using GoTaq® Probe.

TRIP-Br1 (Forward: 5’-GGCCTCTAGCTCCCTCTTT-3’/Reverse: 5’-CGAGCTTGCCAGTAAGTTGT-3’), GAPDH (Forward: 5’-TGCACCACCAACTGCTTAGC-3'/Reverse: 5’-GGCATGGACTGTGGTCATGAG-3’). IR (Forward: 5’-TCCTCAAG AGCTGGAGGAGT-3’/Reverse: 5’-GCTGCTGTCACATTCCCCA-3’), IGF1R (Forward: 5’-TTAAAATGGCCAGAACCTGAG -3'/Reverse: 5’-ATTATAACCAAGCCTCCCAC-3’).

**Immunoprecipitation (IP).** Cells were lysed in NP-40 lysis buffer (Cat #BA1049; Elpis Biotech) supplemented with a protease inhibitor cocktail for 20 min at 4°C. The lysate was centrifuged and the supernatant was incubated with the respective antibodies at 4°C overnight on a Rotospin (SLRM-2M; Mylab Intelli Mixer), followed by incubation with Protein G- or A-agarose beads (Cat#sc-2003; Santa Cruz Biotechnology) for 4 h at 4°C. The samples were then centrifuged and washed with NP40 lysis buffer. The proteins were eluted from the washed beads by boiling for 5 min in 4× SDS gel loading dye and were subjected to immunoblot analysis. Normal mouse IgG (Cat#sc-2025; Santa Cruz Biotechnology) and normal rabbit IgG (Cat#sc-2027; Santa Cruz Biotechnology) were used as controls. To analyze IGF1R and IR ubiquitination, cells were treated with 10 μM MG132 and/or 25 μM CQ for 24 h. The cells were lysed with RIPA buffer containing a protease inhibitor cocktail and centrifuged to obtain cytosolic proteins. Ubiquitinated IGF1R and IR were immunoprecipitated using IGF1R (Cat#ab39675; Abcam) or IR (Cat#3025S; Cell Signaling) antibodies, followed by immunoblotting with Ub antibody (Cat#sc-8017; Santa Cruz Biotechnology).

**Immunofluorescence (IF).** Cells were plated at a density of 5 × 10^4^ cells in a confocal dish (Coverglass-Bottom Dish, Cat#100350; SPL) for 24 h. The cells were fixed in 4% paraformaldehyde for 15 min and washed three times with PBS. The cells were blocked with IF blocking buffer (PBS, 2% BSA, 0.3% Triton-X 100) for 1 h and stained with NEDD4-1 (Cat#sc-25508; Santa Cruz Biotechnology), IGF1R (Cat#sc-81464; Santa Cruz Biotechnology), and IR (Cat#3025S; Cell Signaling) in IF buffer for 12 h. Alexa Fluor^®^ 568 (Cat#ab150115; Abcam) and Alexa Fluor^®^ 647 (Cat#ab175473; Abcam) were diluted 1:100 in IF blocking buffer. The nuclei were stained with DAPI (Cat#P36931; Invitrogen) for 10 min after washing with PBS. Confocal images were obtained using a Zeiss confocal microscope (A1 confocal microscope; Nikon).

**Immunohistochemical (IHC) analysis.** The mouse organs were dissected and fixed in 4% formalin overnight at room temperature. The samples were washed with PBS and incubated in 70%, 80%, 95%, and 100% ethanol for 30 min each. They were then transferred to xylene for 4 h, embedded in paraffin, cut into 5-μm sections using a microtone, dried at room temperature, and deparaffinized at 60°C for 1 h. The slides were then incubated in 10 mM sodium citrate buffer (pH 6.0) at 100°C for 20 min, cooled, and then washed with distilled water for 5 min. Peroxidase activity was removed in methanol buffer containing 0.3% H_2_O_2_, washed three times with PBS for 5 min, and blocked with 5% BSA in PBS-T for 1 h at room temperature. The primary antibodies were diluted in blocking solution and used to incubate the sections overnight at 4°C. The following day, the slides were washed with PBS three times and incubated with a specific biotinylated secondary antibody at room temperature for 1 h. Color was developed using ABC KIT (Cat#pk-4000; VECTASTAIN^®^ABC). Finally, the slides were dehydrated and covered using a mounting medium.

**Animal experiments.** TRIP-Br1 knockout mice (RRID: MGI:4437096) with a C57BL/6 genetic background were kindly gifted by Dr. Huang (Hong Kong University of Science and Technology, Hong Kong, China). The mouse strains were genotyped using a PCR assay as described previously [4]. Insulin-deficient mice (C57BL/6-Tg(pH1-siRNAinsulin/CMV-hIDE)Korl) (5-week-old) were purchased from Laboratory Animal Resource Bank (https://lareb.nifds.go.kr/). Mouse embryo fibroblasts (MEF) were isolated as follows: the embryos were dissected and decapitated from 13.5-day pregnant mice bearing wild-type or knockout TRIP-Br1, in which internal organs were removed. Then, the tissues were washed with cold PBS, cut into pieces, and incubated with trypsin/EDTA. Lastly, the cells were transferred to DMEM supplemented with 10% FBS after each incubation. These cells were maintained in DMEM after the removal of the non-adherent cells after 2 h.

**Xenograft study.** Exponentially growing MCF7^WT-TRIP-Br1^ and MCF7^KD-TRIP-Br1^ cells (1 × 10^7^ cells) were collected and re-suspended in 0.1 ml of PBS. The cells were then subcutaneously injected into 5-week-old female null mice. The volumes of the resulting tumors were calculated as follows: 0.523 × length × width^2^. The mice were sacrificed after one month and their tumor weights were measured after resection.

**Single-cell RNA sequencing analysis.** Single-cell RNA-seq data for 11 breast cancer patients were downloaded from GEO (SRP066982). Sequencing reads from raw fastq were aligned using STAR v2.7.8a with the human reference genome GRCh38 and the Genecode.v38 annotations, and transcript per million (TPM) values were estimated using RSEM v1.3.1, with the following options: star--paired-end--estimate-rspd--single-cell-prior. The log2-tansformed TPM value, log2(TPM + 1), was considered as the relative expression level of each gene. Before further analysis, cells of reliable quality were collected after cell-type annotation, as defined in the original paper. To evaluate the association between two factors, we computed Pearson’s correlation coefficient and performed linear regression analysis using the ‘lm’ function from the base package in R. The IR/IGF1R ratio was calculated by subtracting (or dividing) the relative expression of IR and IGF1R. Missing values due to a lack of IGF1R expression were removed from the association analysis.

**Statistics analysis.** Data are presented as the mean ± standard deviation (SD) from three independent experiments. Statistical analysis was performed using Student’s t-test to compare two different groups or one-way analysis of variance followed by Bonferroni's multiple comparisons test to compare multiple groups. SPSS Statistics version 23 (IBM Corporation, Armonk, NY, USA) was used to analyze the data. P < 0.05 was used to denote statistical significance.

# **Supplementary references**

1. Jung S, Li C, Duan J, Lee S, Kyeri Kim K, Park Y, Yang Y , Kim KI, Lim JS, Cheon CI, YS, Lee MS. TRIP-Br1 oncoprotein inhibits autophagy, apoptosis, and necroptosis under nutrient/serum-deprived condition. Oncotarget. 2015;6(30):29060-29075.

2. Hong SW, Kim CJ, Park WS, Shin JS, Lee SD, Ko SG, Jung S, Park IC, An SK, Lee WK, Lee WJ, Jin DH, Lee MS. p34SEI-1 inhibits apoptosis through the stabilization of the X-linked inhibitor of apoptosis protein: p34SEI-1 as a novel target for anti-breast cancer strategies. Cancer Res. 2009;69(3):741-746.

3. Jung S, Li C, Duan J, Lee S, Kim K, Park Y, et al. TRIP-Br1 oncoprotein inhibits autophagy, apoptosis, and necroptosis under nutrient/serum-deprived condition. Oncotarget. 2015;6(30):29060-29075.

4. Pablo J Fernandez-Marcos 1, Cristina Pantoja, Agueda Gonzalez-Rodriguez, Nicholas Martin, Juana M Flores, Angela M Valverde, Eiji Hara, Manuel Serrano. Normal proliferation and tumorigenesis but impaired pancreatic function in mice lacking the cell cycle regulator sei1. PloS One. 2010;5(1):e8744.
